# A cross‐scale transfer learning framework: prediction of SOD activity from leaf microstructure to macroscopic hyperspectral imaging

**DOI:** 10.1111/pbi.14566

**Published:** 2025-01-09

**Authors:** Jie Hao, Yan Yan, Yao Zhang, Yiyang Zhang, Yune Cao, Longguo Wu

**Affiliations:** ^1^ School of Wine & Horticulture Ningxia University Yinchuan Ningxia China; ^2^ College of Mechanical and Electronic Engineering, Northwest A&F University Yangling Shaanxi China; ^3^ State Key Laboratory of Vegetable Biobreeding, Institute of Vegetables and Flowers Chinese Academy of Agricultural Sciences Beijing China; ^4^ College of Animal Science and Technology, Ningxia University Yinchuan Ningxia China; ^5^ Key Laboratory of Quality and Safety of Wolfberry and Wine for State Administration for Market Regulation Institute of Food Testing in Ningxia Yinchuan Ningxia China; ^6^ Ningxia Modern Protected Horticulture Engineering Technology Research Center Yinchuan Ningxia China

**Keywords:** superoxide dismutase, microhyperspectral imaging technology, heterogeneous two‐dimensional correlation spectra, CNN‐LSTM algorithm, transfer learning

## Abstract

Superoxide dismutase (SOD) plays an important role to respond in the defence against damage when tomato leaves are under different types of adversity stresses. This work employed microhyperspectral imaging (MHSI) and visible near‐infrared (Vis–NIR) hyperspectral imaging (HSI) technologies to predict tomato leaf SOD activity. The macroscopic model of SOD activity in tomato leaves was constructed using the convolutional neural network in conjunction with the long and short‐term temporal memory (CNN‐LSTM) technique. Using heterogeneous two‐dimensional correlation spectra (H2D‐COS), the sensitive macroscopic and microscopic absorption peaks connected to tomato leaves' SOD activity were made clear. The combination of CNN‐LSTM algorithm and H2D‐COS analysis was used to research transfer learning between microscopic and macroscopic models based on sensitive wavelengths. The results demonstrated that the CNN‐LSTM model, which was based on the FD preprocessed spectra, had the best performance for the microscopic model, with R_C_ and R_P_ reaching 0.9311 and 0.9075, and RMSEC and RMSEP reaching 0.0109 U/mg and 0.0127 U/mg respectively. There were 10 macroscopic and 10 microscopic significant sensitivity peaks found. The transfer learning was carried out using sensitive wavelengths, and the model performed well with an R_P_ value of 0.7549 and an RMSEP of 0.0725 U/mg. The combined CNN algorithm and H2D‐COS analysis demonstrated the viability of transfer learning across microscopic and macroscopic models for quantitative tomato leaf SOD prediction.

## Introduction

As one of the largest horticultural crops cultivated in China's facilities, tomato has become a major economic pillar crop in many regions. Tomatoes, containing lycopene, vitamin C, minerals and other nutrients, have a high food value (Sun *et al*., 2021). Tomato is susceptible to a variety of adversity stresses during natural growth, such as drought, salinity, high temperature, low temperature, pests and diseases. During the growth of tomatoes, salt in the soil is one of the limiting factors that can affect the normal healthy growth and the quality production of tomatoes (Singh *et al*., 2015). Under normal development circumstances, the organism of the plant balances the generation and elimination of reactive oxygen compounds (Bankaji *et al*., 2019). In the case of salt stress, the balance is broken. Because the plant cannot fend against salt stress via its defensive systems, too many reactive oxygen species build up inside the plant, which results in oxidative stress (Acosta‐Motos *et al*., 2017). The antioxidant system of plants is self‐operating to defend the organism from stressful conditions. Superoxide dismutase (SOD), catalase (CAT) and peroxidase (POD) are three antioxidant enzymes that are crucial. SOD catalyses the disproportionation reaction of O^2−^ to produce H_2_O_2_ and O_2_, and H_2_O_2_ is subsequently scavenged by POD and CAT (Ashraf *et al*., 2018). When subjected to adversity stress, tomato leaves show obvious physiological and phenotypic changes, such as leaf yellowing, wilting and the appearance of lesions. All these changes are closely related to the activity and distribution of SOD, which enables researchers to analyse the role of SOD in adversity stress more intuitively by observing the appearance of leaves and detecting physiological indicators (Mehlmer *et al*., 2010). Physicochemical analysis is the primary method used in the conventional approach to identify intracellular antioxidant enzymes in leaves (Taniguchi *et al*., 2022). During the experiment, these techniques need a lot of time and reagents. In the meanwhile, current techniques are unable to identify tomato leaves quickly in situ online, nor can they disclose the intracellular spatial distribution or dynamic change processes and mechanisms (Peskin and Winterbourn, 2017; Wu and Chen, 2019). Therefore, it is urgently necessary for a rapid and nondestructive visualization method to detect intracellular antioxidant enzymes in leaves.

HSI technique is frequently used to detect crops both quantitatively and qualitatively at the macroscopic level (Bai *et al*., 2020; Zhou *et al*., 2022). When it comes to compounds with low component content and smaller structures, conventional HSI is less sensitive to their spectra. Using traditional HSI, SOD detection was primarily limited to macroscopic leaf areas. This results in a weak direct link between SOD properties and spectral imaging features, making it challenging to identify minute alterations in the material within cells. Dynamic variations of microscopic substances inside cells inevitably cause variations of spectral curves in terms of wave shape and peak value (Zarco‐Tejada *et al*., 2012). Under salt stress adversity, microhyperspectral imaging (MHSI) study can show the heterogeneity of SOD distribution in leaf cells at the cellular level (Lin *et al*., 2019). Kang *et al*. (2020) proposed a combination of MHSI technique and a hybrid deep learning framework for rapid classification of foodborne bacteria at the single‐cell level and obtained more reliable results with accuracy values up to 98.40%. Qin *et al*. (2020) used MHSI to investigate the visualization of the distribution of microstructures of matcha particle size. However, the complexity of slice creation, the destructive nature of the sample and the difficulty in determining the physicochemical parameters of the scanned area, especially in determining the chemical values in microregions lead to the complicated of direct experimental manipulation using MHSI (Rodrigues *et al*., 2018). Resolution of complex MHSI experiment and difficult modelling has become a challenge in our group's research.

In recent years, deep learning has solved many complex pattern recognition and regression prediction problems in many fields. (Al‐Sarayreh *et al*., 2020; Barbon *et al*., 2016; Ma *et al*., 2020; Qin *et al*., 2020). As a type of deep feed‐forward neural network with local connection and weight sharing, convolutional neural networks (CNNs) are the most well‐known of them (Hao *et al*., 2022; Qi *et al*., 2020; Zhang *et al*., 2020). Meanwhile, memory units in long‐ and short‐term temporal memory (LSTM) make neural networks more suitable for time series analysis and modelling, and can acquire information in time series more efficiently (Zhang and Li, 2022). The two neural network models are integrated after taking into account their respective benefits. Zhang *et al*. (2023) developed a prediction model for moisture content of oilseed rape leaves using CNN‐LSTM model with coefficient of determination and root mean square error for the prediction set (RMSEP) of 0.814 and 0.005 respectively. Zhang *et al*. (2022) used partial least squares regression (PLSR), CNN, LSTM and CNN‐LSTM to develop a prediction model for moisture content of single kernel maize, and the results showed that the CNN‐LSTM model was optimal. This indicates that CNN‐LSTM has full potential in predicting SOD activity in tomato leaves.

Transfer learning is the process of applying information from the source domain to a new task in the target domain (Zhang *et al*., 2021). Most models need to be rebuilt from scratch using newly collected training data when observational conditions such as time, location and scale change. In addition to this, a large number of labelled samples are required to ensure the stability of both types of models (Zhang *et al*., 2021). However, in practical application scenarios, this approach is costly and has low feasibility. Therefore, transfer learning is used to make predictions for new scenarios (time, place, sensors, scales, etc.) in order to achieve a reduction of the effort for new data collection (Zhou *et al*., 2023). Transfer learning is a new learning paradigm in machine learning that is generally used for information transfer between different domains. Through the similarity between tasks, the existing detection model is applied to the learning process of a new detection task, thus solving the problem of insufficient data and improving the generalization ability. (Zhou *et al*., 2024). By either employing the information gleaned from the pertinent data or choosing some unlabelled data to be labelled, transfer learning speeds up the training process (Wang *et al*., 2021). The field of remote sensing has effectively used transfer learning, which has shown effectiveness in managing variations in the spectrum and spatial data of the same HSI obtained by the same sensor at several periods or locations (Le *et al*., 2020). To solve the complex experiments and difficult modelling of MHSI, transfer learning based on CNN‐LSTM algorithm was proposed. This study used a small number of target samples for prediction and CNN‐LSTM in conjunction with transfer learning to train the target data using auxiliary source data. The micro‐spectral model was migrated to macro‐spectra to implement micro‐spectral and macro‐spectral correlation.

Heterogeneous two‐dimensional correlation spectroscopy (H2D‐COS) has been used to study the correlation between different types of wavebands, and to identify the complementarity of two spectra with varying components, by combining two different types of spectra into a single spectrum (Cheng *et al*., 2018; Tang *et al*., 2019). By connecting two entirely distinct types of spectral wavelengths recorded under the same perturbation, H2D‐COS can shed light on the spectral peak assignment (Tang *et al*., 2019). H2D‐COS has become one of the most dynamic research areas in 2D‐COS (Maqbool *et al*., 2020). Therefore, H2D‐COS was utilized to find the wavelengths in which microscopic and macroscopic spectral variations were correlated with each other to establish migration relationships.

This study used a combination of H2D‐COS and transfer learning based on the CNN‐LSTM algorithm to predict SOD activity in tomato leaves under various salt stress scenarios. The goal of this paper is to achieve the transfer learning between MHSI and Vis–NIR HSI, that is, between microscopic and macroscopic HSI. Based on the microscopic quantitative research of SOD in tomato leaves, we explored the model transfer learning from microscopic to macroscopic SOD activity in tomato leaf cells. We applied H2D‐COS analysis to seek microscopic and macroscopic sensitive absorption peaks associated with variations in SOD activity of tomato leaves. Based on the microscopic and macroscopic sensitive absorption peaks, CNN‐LSTM‐based transfer learning was used to implement model migration for both microscopic and macroscopic HSI. The results of this study were expected to provide a reference for future studies on microscopic trace substance monitoring.

## Results and discussion

### Property characterization statistics

**Figure 1:**
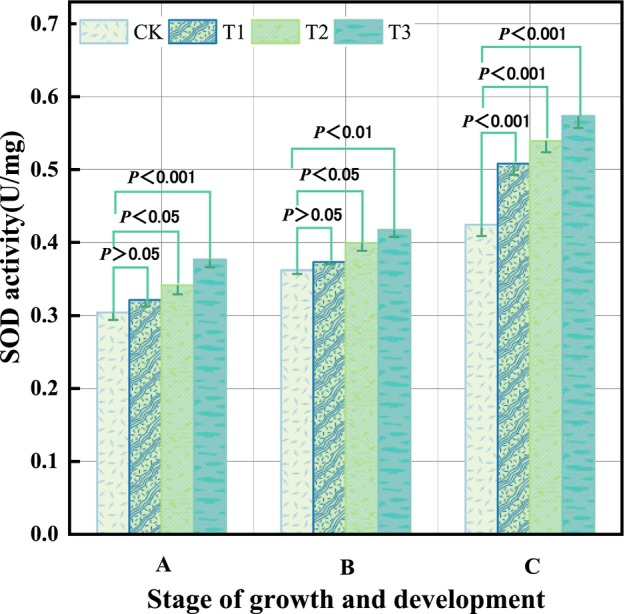
The outcomes of variations in SOD activity levels in tomato leaves. Stages A, B and C represent the tomato seedling, flowering and fruit‐setting phases respectively. Lowercase letters a, b, c and d indicate the results of statistically significant difference.

The findings of the variations in tomato leaf SOD activity levels depending on the treatment circumstances of various concentrations of salt solutions during various fertility phases are displayed in Figure 1. During the various stages of tomato growth and development, the SOD activity of tomato leaves was considerably increased with an increase in salt content. SOD activity under the T1 treatment approach was not significantly different from the control at the tomato seedling stage. This was probably attributed to the fact that the tomato plants were in the early stages of growth reacting less to low concentrations of salt solution. The other two treatments showed significant differences in tomato leaf SOD activity compared to the control. Higher salt solutions caused internal regulatory mechanisms to be activated in tomato leaves to achieve protection in response to salt stress. Higher salt solutions caused internal regulatory mechanisms to protect tomato leaves in response to salt stress. Significant variations in SOD levels were observed between the control and other three treatments during the tomato blooming and fruiting stages. Under the three different concentrations of salt stress, the SOD activity was significantly enhanced with increasing concentrations. During the tomato fruiting stage, there were significant differences in SOD activity values for different levels of salt stress, that is, the SOD values increased continuously with increasing salt concentration. The SOD activity levels of tomato leaves rose with tomato development and the length of the salt stress over the whole tomato reproductive phase, reaching its maximum value at the fruiting stage. Thus, the SOD activity of tomato seedling leaves had a dominant increase with the accumulation of both concentration and time.

### Spectral curve analysis

#### Microhyperspectral curve analysis

**Figure 2:**
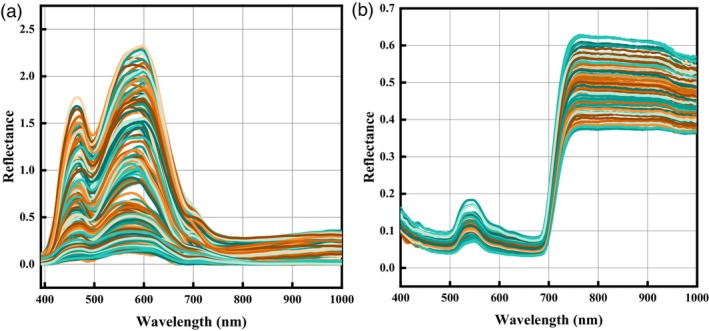
The curves of tomato leaves. (a) MHSI curves; (b) Vis–NIR hyperspectral curves.

The microscopic spectral curves obtained for the tomato samples are shown in Figure 2a. The original MHSI curve was consistent with the trend of the average MHSI at different fertility periods. The spectral reflectance of tomatoes varied considerably from one reproductive stage to another. This was caused by the fact that with the increase of irrigation time in salt solution and the plant cell structure was damaged, the plant cells will protect themselves through the antioxidant system. Two absorption peaks at 480 nm and 560 nm and a valley at 500 nm were found. The curve was minimized and levelled off at around 700 nm. Visible light's green reflection area was found to correlate with the MHSI peak at 560 nm. Carotenoids and chlorophyll pigments were shown to absorb red light in the valley at 690 nm (Cui *et al*., 2020).

#### Vis–NIR hyperspectral curve analysis

The Vis–NIR hyperspectral curves are displayed in Figure 2b. Around 545 nm, there are two absorption dips and one absorption peak on the spectral curve. These peaks and valleys may be due to a combination of OH bonds, water, and tomato leaf surface colour (Xiang *et al*., 2022). It is possible to more accurately reflect the green surface of a tomato leaf by using visible light with a colour spectrum of yellow to green The reflectance of the spectrum is relatively high between 755 nm and 900 nm. The small valley at 950 nm was probably associated with the second and first overtones of the O‐H stretch (Qin *et al*., 2020). When plants are exposed to salt stress, their ability to photosynthesize is strongly correlated with the wavelength range of 495–680 nm. In reaction to this stress, the plants' own internal regulatory systems begin to function (Zhang *et al*., 2013).

### Microscopic hyperspectral modelling

Figure S1 shows the microhyperspectral curves pretreated by smoothing, baseline and FD methods. Figure S1a,b show the original spectral curves and the spectral curves after smoothing preprocessing, and the two curves were similar. Figure S1c shows the spectral curves preprocessed by baseline method. In the front part of the curve, the spectra were similar to the original spectra and in the back part of the curve the spectra were normalized. Figure S1d shows the spectral curves processed by FD method, the curves were normalized and the sensitive absorption peaks were intensified to amplify the differentiation of the curves. The model performance of the hyperspectral with different preprocessing is shown in Figure S2. There was no significant difference between the CNN‐LSTM models developed by the original spectra and the spectra after smoothing preprocessing. This was likely due to the similarity between the original and smoothing preprocessed spectral curves. Compared to the original spectra and smoothing approach, the model constructed by the spectra following baseline pretreatment performed better. The CNN‐LSTM model constructed using the FD preprocessed spectra was the best model after integrating the findings of the calibration and prediction sets; RC and RP reached 0.9311 and 0.9075, and RMSEC and RMSEP reached 0.0109 and 0.0127 U/mg respectively. This indicated that the peaks of the FD‐pretreated spectral curves were identified and correlated with the SOD activity of the tomato leaves. Therefore, the processed data by FD method were selected as the basis for the subsequent analysis.

Kong *et al*. (2014) employed HSI for rapid detection of POD activity in tomato grey grapevine infected leaves and the results showed good performance with R_P_ and RMSEP of 0.8647 and 465.9880 respectively. Zhu *et al*. (2020) applied polarized spectral‐hyperspectral data fusion techniques for the nondestructive diagnosis of soluble sugars, total nitrogen and their ratios in tomato leaves in the greenhouse, and the results showed that the polarized spectral‐hyperspectral multidimensional information detection method can effectively determine the nutrient stress conditions in tomato. Fang *et al*. (2012) developed the HSI model for the prediction of SOD activity in tomato leaves, which reached RP and RMSEP of 0.9353 and 37.80 U/g respectively. The above studies have demonstrated the great potential of HSI for predicting the microscopic substances in tomato leaves. However, there has not been any study done on finding SOD activity in tomato leaves thus far. In this paper, HSI was used to detect the SOD activity of tomato leaves, which is one of the innovations of this paper.

### Heterogeneous two‐dimensional correlation spectral analysis

**Figure 3:**
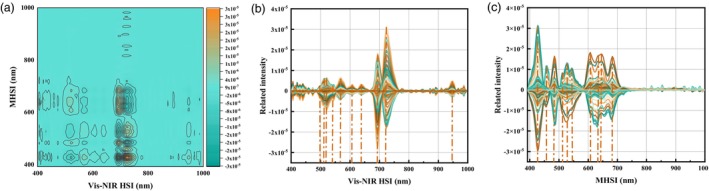
The H2D‐COS and slice spectra constructed by Vis–NIR HSI and MHSI. (a) The H2D‐COS of Vis–NIR HSI variations induced by MHSI; (b) The slice spectra of Vis–NIR HSI; (c) The slice spectra of MHSI.

Tomato leaves are a system containing many kinds of complex substances. This made the microscopic and macroscopic spectra of tomato leaves extremely complex, and it was difficult to clarify the correlation between microscopic and macroscopic spectral peaks. The weak peaks and overlapping peaks on the original 1D spectrum were sharpened to improve the spectral resolution by 2D‐COS analysis. According to the analysis in Section 3.3, it was found that the modelling performance of microscopic spectra based on FD preprocessing method performed the best. The FD preprocessed spectral data were employed to construct H2D‐COS for the analysis. Figure 3 shows the H2D‐COS constructed by Vis–NIR HSI and MHSI. Figure 3a shows the H2D‐COS of the Vis–NIR HSI variations induced by MHSI, that is, the H2D‐COS of the MHSI variations induced by Vis–NIR HSI. The correlated absorption peaks can be observed, but the specific location of the sensitive peaks was rather ambiguous. Slice spectra were generated to clarify the variations of Vis–NIR HSI sensitive absorption peaks caused by MHSI, which are shown in Figure 3b. The peaks at the initial and final ends were excluded because of the instability of the instrument during the collection of hyperspectral images, resulting in the instability of the initial and final ends of the curve. The peak at 948 nm was identified by H2D‐COD analysis as a sensitive peak that may be associated with variations of SOD activity in tomato leaves. In addition to this, it is clear from the spectral curves that the data above 750 nm do not seem to provide any significant information. This phenomenon was also confirmed by the H2D‐COS analysis. A total of 10 relevant sensitive peaks were found at 497, 511, 516, 540, 564, 607, 641, 694, 723 and 948 nm. Figure 3c shows the corresponding slice spectra. A total of 10 relevant sensitive peaks were observed at 426, 458, 481, 510, 527, 547, 607, 631, 641 and 682 nm. The absorption peaks sensitive to Vis–NIR HSI variations induced by MHSI and the absorption peaks of MHSI induced by Vis–NIR HSI may be sensitive peaks related to the SOD activity of tomato leaves extracted by 2D‐COS. Therefore, the sensitive peaks of the two spectral responses were correlated to model transfer and quantify the SOD activity in tomato leaves. The analytical results also showed that the data above 750 nm are hardly considered.

In previous studies, H2D‐COS has been used to detect correlations between two spectral signals from the response patterns of two systems. Cheng *et al*. (2018) used H2D‐COS to correlate near‐infrared (NIR) HSI data with mid‐infrared spectroscopy to identify characteristic correlation bands and to develop a model for monitoring oxidative damage in pork myogenic fibres. Yang *et al*. (2016) constructed H2D‐COS using infrared and NIR spectra to explore the correlation between the two spectra and to develop the adulterated milk discrimination model and obtain good discrimination accuracy. Takashi *et al*. (2013) used online NIR and mid‐infrared (MIR) dual‐zone spectrometry in conjunction with H2D‐COS of NIR and MIR to locate bands of high positive correlation intensity with ethanol concentration during fermentation. These bands were then utilized to develop a regression model. The expected outcomes show that there is a good match between the ethanol concentrations determined by high‐performance liquid chromatography and those determined using PLS regression models. The above studies have shown that the method of constructing H2D‐COS using two types of spectra is a tool for seeking sensitive bands with strong correlation with the object to be measured, and an effective method for building reliable regression models. Therefore, the sensitive peaks associated with SOD activity in tomato leaves obtained by correlating Vis–NIR HSI and MHSI by applying H2D‐COS in this paper can be applied in subsequent analyses.

### Transfer learning

**Figure 4:**
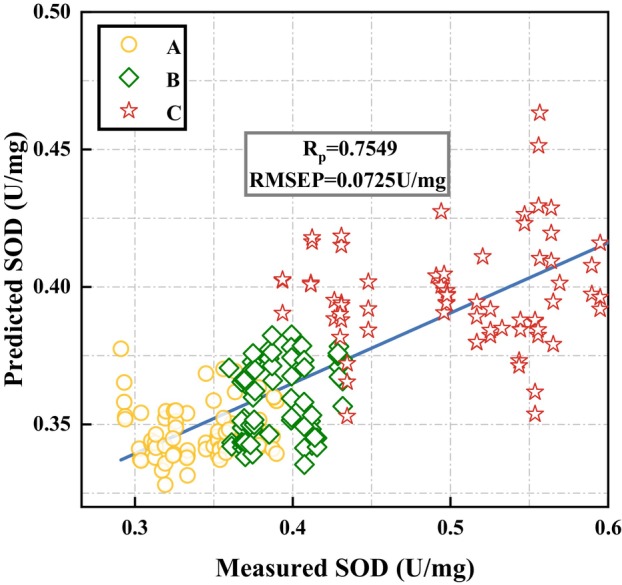
The transfer model performance. Stages A, B and C represent the tomato seedling, flowering and fruit‐setting phases respectively.

Based on the research of microscopic quantitative model of SOD in tomato leaves, we explored the model transfer learning from microscopic to macroscopic SOD activity in tomato leaf cells. The microscopic spectral features of tomato leaf SOD were transferred to macroscopic spectra for analysis by correlating and transferring sensitive peaks. The transfer from microscopic to macroscopic spectral features of tomato leaf SOD was accomplished by correlating sensitive peaks that may be related to the SOD activity of tomato leaves according to the analysis in Section 3.4. Figure 4 shows the transfer model performance, with R_P_ reaching 0.7549 and RMSEP reaching 0.0725 U/mg. There were differences between the characteristics of macroscopic spectra and microscopic spectra. However, the transfer model performance showed that the relevant sensitive peaks extracted by H2D‐COS method were strongly correlated with the SOD activity of tomato leaves. Therefore, it is feasible to combine CNN‐LSTM with H2D‐COS for quantitative model transfer of tomato leaves. This can replace complex microspectral modelling in future studies.

Xiao *et al*. (2022) combined spectral preprocessing with deep transfer learning for assessing the chlorophyll content of cotton leaves to achieve the migration between different varieties of cotton leaf chlorophyll content, and the migration model achieved good research results. Wan *et al*. (2022) combined transfer learning and hyperspectral reflectance to enable the assessment of leaf nitrogen concentrations across different plants. Zhang *et al*. (2021) suggested using transfer learning in conjunction with amplitude‐ and shape‐enhanced two‐dimensional correlation spectroscopy (2DCOS) to monitor the chlorophyll content of leaves in winter wheat. They also took into account the connection between external perturbations and hyperspectral amplitude and shape features to improve the dynamic spectral response. The findings demonstrated the method's excellent accuracy and resilience, as well as its applicability to the identification and inversion of other important agricultural factors. In previous studies on transfer learning of plant leaves, most of them are about transfer learning between different species of the same plant and different plants. Compared with previous studies, in this study, H2D‐COS combined with deep learning was used to explore the microscopic and macroscopic peaks that are highly sensitive to the changes of SOD activity in tomato leaves for the first time, and transfer learning between two devices, MHSI and Vis–NIR HSI, was realized.

### Visualization

**Figure 5:**
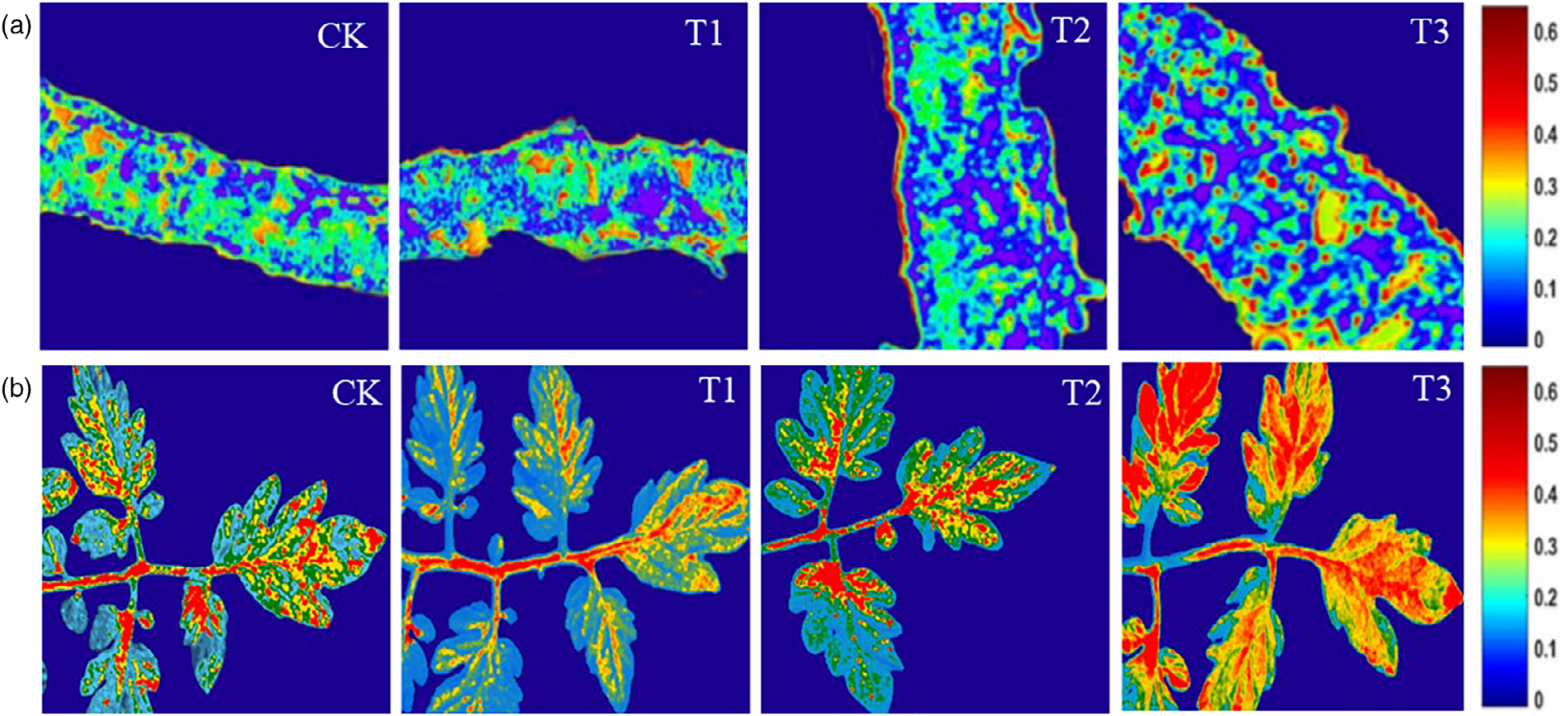
The visualization of SOD distribution in tomato leaves. (a) Microscopic prediction model; (b) Macroscopic prediction model.

The visualization of SOD activity in tomato leaves was based on the best microscopic and microscopic prediction models to clarify the distribution of SOD activity in cells in the microscopic domain. The distribution of SOD activity values corresponding to the pixels was indicated by the variation of different colours and the shades of the colours. The gradual shift of colours from blue to red in the image indicated the change of SOD activity values from low to high. Figure 5 reflects the distribution of SOD activity in the tomato leaves under different treatment conditions, where the magnitude of the values reflected in the chromatic band was consistent with the spectral reflectance values under each period. As salt stress intensified, SOD activity in tomato leaves increased, accompanied by an increasing distribution of SOD in tomato leaves. With the increase of salt concentration, the SOD distribution of tomato leaves increased significantly and the colour of the visualization maps gradually deepened from the initial blue to red. This demonstrated a significant increase in the distribution of SOD activity values of tomato leaves, reaching maximum activity values when tomato plants reached the fruiting stage. Therefore, transfer learning can not only accomplish rapid monitoring of tomato leaves SOD activity but also provide visualization maps.

## Conclusion

In this study, H2D‐COS analysis combined with transfer learning based on CNN‐LSTM algorithm was proposed to investigate the SOD activity of tomato leaves. From the upper, middle and bottom levels of the tomato plants at various fertility times, a total of 324 tomato leaves were taken. Comparing the modelling results of different preprocessing methods, the CNN‐LSTM model for microscopic HSI established based on the FD pretreated spectra performed the best, with R_P_ and PMSEP reaching 0.9075 and 0.0127 U/mg respectively. According to the results of the H2D‐COS analysis, 10 Vis–NIR HSI sensitive absorption peaks were observed, including 497, 511, 516, 540, 564, 607, 641, 694, 723 and 948 nm, and 10 MHSI sensitive absorption peaks were 426, 458, 481, 510, 527, 547, 607, 631, 641 and 682 nm. The sensitive peaks of the two spectral responses were correlated to model transfer and quantify the SOD activity in tomato leaves. The transfer model performed well with an R_P_ value of 0.7540 and an RMSEP of 0.0725 U/mg. The findings showed that a quick and incredibly accurate microscopic and macroscopic prediction model of tomato leaf SOD activity could be established using the H2D‐COS matrix created by MHSI and Vis–NIR‐HSI. Transfer learning between microscopic and macroscopic hyperspectral was achieved for the first time, providing novel methods and concepts for the observation and quantification of SOD activity in tomato leaves. This study laid the foundation for the quantitative detection of trace substances in tomato leaves and the application of H2D‐COS in transfer learning.

## Materials and methods

### Sample preparation

The seedlings were placed in substrate culture after being moved when the cotyledons unfurled into three to four pieces. Tomato plants were irrigated with four salt solutions made with NaCl at varying concentrations (0 g/L (CK), 1 g/L (T1), 2 g/L (T2) and 3 g/L (T3)) once the slowing phase was over. For every variation in concentration, 27 plants were collected, making 108 total. Using the tomato plants at several stages of fertility, 324 tomato leaves in total were taken from the top, middle and bottom levels. Scanning of the spectra and preparation of the slices were performed.

### SOD activity measurement

The nitrogen blue tetrazolium photochemical reduction technique was used to determine the SOD activity. First, roughly 300 mg of tomato leaves was measured out in a mortar. One millilitre of phosphate buffer was then added, and the mixture was ground (100 mmol/L, pH = 7.8). After grinding into a slurry, the slurry was then rinsed with 1 mL of phosphate buffer and mixed. The enzyme solution was identified in the supernatant after the mixture was poured into a centrifuge tube and centrifuged at 4 °C. After 0.05 mL of enzyme solution was added to the reaction system, it was illuminated at 25 °C for 25 min. A blank consisting of 3 mL of nitrogen blue tetrazolium (NBT) solution (0.750 mmol/L) and 0.05 mL of buffer solution was also used as a control, and the buffer solution was used as a zeroing cup. Absorbance values at 560 nm were measured accurately. The inhibition of NBT photochemical reduction by 50% was taken as one unit of enzymatic activity. Using Equation 1, the SOD enzyme activity of a tomato leaf was determined.

(1) $$\mathrm{SOD}\left(U/\mathrm{mg}\right)=\frac{\left(A_{\mathrm{So}}-A_{S}\right)\times V_{t}}{A_{\mathrm{So}}\times W\times V_{S}\times 0.5\times 1000}$$

where *A*_*S0*_ is the absorbance value of control tube under light, *A*_*S*_ is the absorbance value of sample determination tube, *V*_*t*_ is the total volume of sample extract, *V*_*S*_ is the volume of extract at determination and W is the fresh weight of sample.

### Hyperspectral image acquisition

The MHSI system integrated the hyperspectral system with the microscope. Therefore, along with acting as a hyperspectral system, the MHSI system may also enter the cell microregion for microscopic detection by combining the function of a microscope's objective lens and optical path. Meanwhile, we took into account the difference in focal depth between normal HSI and MHSI. Vis–NIR HSI can obtain reflectance spectra of SOD activity variations in tomato leaves. MHSI is microscopic imaging combined with Vis–NIR HSI technology. Although HSI and MHSI are different in focal depth, the imaging principle is the same. Compared to HSI, the beam of MHSI can be focused on the micrometre scale region, which is characterized by micro‐trace detection and high sensitivity. Dynamic variations of microscopic substances in cells inevitably lead to variations in the spectral profile and waveform peaks. Therefore, MHSI analysis is more capable to identify physiological variations in SOD activity before and after adversity stress at the microscopic level, and can reveal variations in SOD distribution in leaf cells under salt stress adversity at the cellular level. Therefore, transfer learning based on CNN‐LSTM algorithm was combined with H2D‐COS for migration of macro‐ and microdevices.

Prior to obtaining the spectral picture, black and white correction and scanning parameters must be completed in order to produce a clean and distortion‐free image. Equation 2 displays the adjustment in black and white.

(2) $$R=\frac{\mathrm{Ro}-D}{W-D}$$

where *R*_*0*_ is the original image, *D* is the black image, *W* is the white image and R is the calibrated hyperspectral image. The experimental settings included an exposure length of 11 ms with a gain of 2, a starting position of 0.65 cm, a scanning distance of 1.0 cm and scanning speeds of 0.8 and 0.2 cm/s, respectively, for the forward and backward scans.

### Hyperspectral data preprocessing

Smoothing, baseline, and first‐order (FD) derivative were among the spectrum preprocessing techniques used in this work to improve the spectra. In the preceding paper, the experimental mathematical models and the concepts of the preprocessing algorithms were thoroughly presented (Biswas *et al*., 2016; Ramos *et al*., 2022).

### Heterogeneous two‐dimensional correlation spectral analysis

2D‐COS is the variation of spectral intensity *y*(*v*, *p*) considering external disturbance variables (e.g. pressure, concentration, electric field, magnetic field, etc.), where *v* is the spectral variable and *p* is the external disturbance variable (Cheng *et al*., 2018; Kim *et al*., 2006). For the dynamic spectrum $\tilde{y}$(*v*, *p*) induced by the system in a certain external disturbance interval (1 ~ *N*) is defined.

(3) $$\tilde{y}\left(v,p\right)=\left\{\begin{array}{c} y\left(v,p\right)-\bar{y}\left(v\right)\;1\leq p\ll N\\ 0\;\text{otherwise} \end{array}\right.$$

$\bar{y}$(*v*) is the reference spectrum of the system, which is usually elected as the average spectrum. The reference spectrum is defined by Equation 4.

(4) $$\bar{y}\left(v\right)=\frac{1}{N-1}\sum_{j=1}^{N}y\left(v,p_{j}\right)$$

The two‐dimensional simultaneous spectrum is defined as Equation 5.

(5) $$\Phi \left(v_{1},v_{2}\right)=\frac{1}{N-1}\sum_{j=1}^{N}\tilde{y}\left(v_{1},p_{j}\right)\cdot \tilde{y}\left(v_{2},p_{j}\right)$$

The two‐dimensional asynchronous spectrum is defined as Equation 6.

(6) $$\Psi \left(v_{1},v_{2}\right)=\frac{1}{N-1}\sum_{j=1}^{N}\tilde{y}\left(v_{1},p_{j}\right)\cdot \sum_{k=1}^{N}M_{\mathrm{jk}}\cdot \tilde{y}\left(v_{2},p_{k}\right)$$

where *M*_*jk*_ represents the elements of row *j* and column *k* of the Hilbert–Noda transformation matrix, and is denoted as follows.

(7) $$M_{\mathrm{jk}}=\left\{\begin{array}{c} 0\;\text{if}\;j=k\\ \frac{1}{\pi \left(k-j\right)}\;\text{otherwise} \end{array}\right.$$

The two‐dimensional correlation intensity *X*(*v*_*1*_, *v*_*2*_) represents a comparison of the spectral variables *v*_*1*_ and *v*_*2*_ as a function of the change in spectral intensity $\tilde{y}$(*v*, *p*) in the interval of the external disturbance variables. Since the correlation function calculates the change in intensity at 2 mutually independent spectral variables *v*_*1*_ and *v*_*2*_, *X*(*v*_*1*_, *v*_*2*_) can be transformed into a complex number function.

(8) $$X\left(v_{1},v_{2}\right)=\Phi \left(v_{1},v_{2}\right)+i\Psi \left(v_{1},v_{2}\right)$$

Vis–NIR HSI and MHSI were introduced to construct H2D‐COS based on macroscopic and microscopic spectra. H2D‐COS is a comparison of the dynamic spectrum (*v*, *t*) measured by one technique (Vis–NIR HSI) with the dynamic spectrum (*v*, *t’*) measured by another completely different spectrum (MHSI). The general form of H2D‐COS is defined as Equation 9.

(9) $$X\left(v_{1},v_{2}\right)=<\tilde{y}\left(v,t\right)\tilde{z}\left(v,t^{\prime }\right)>$$

**Figure 6:**
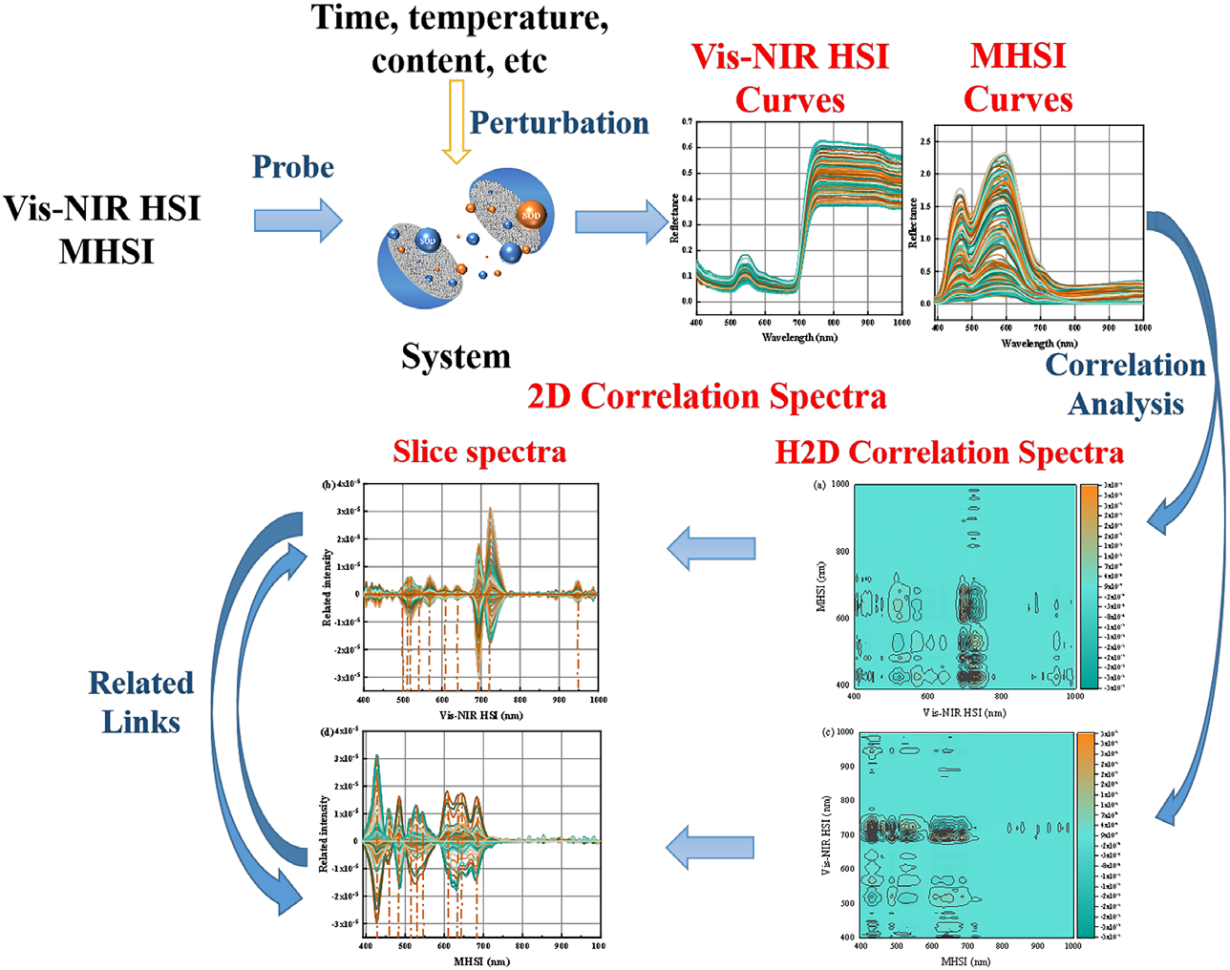
The acquisition process of H2D‐COS.

An illustration of the H2D‐COS creation process is presented in Figure 6. The temporal distributions of the various variables were combined linearly to form the vertical slice of the initial pairwise system. H2D‐COS analysis in combination with slice spectroscopy enables the identification of sensitive peaks with high correlation between macroscopic and microscopic spectra regarding SOD activity for subsequent model migration learning.

### CNN‐LSTM model architecture

**Figure 7:**
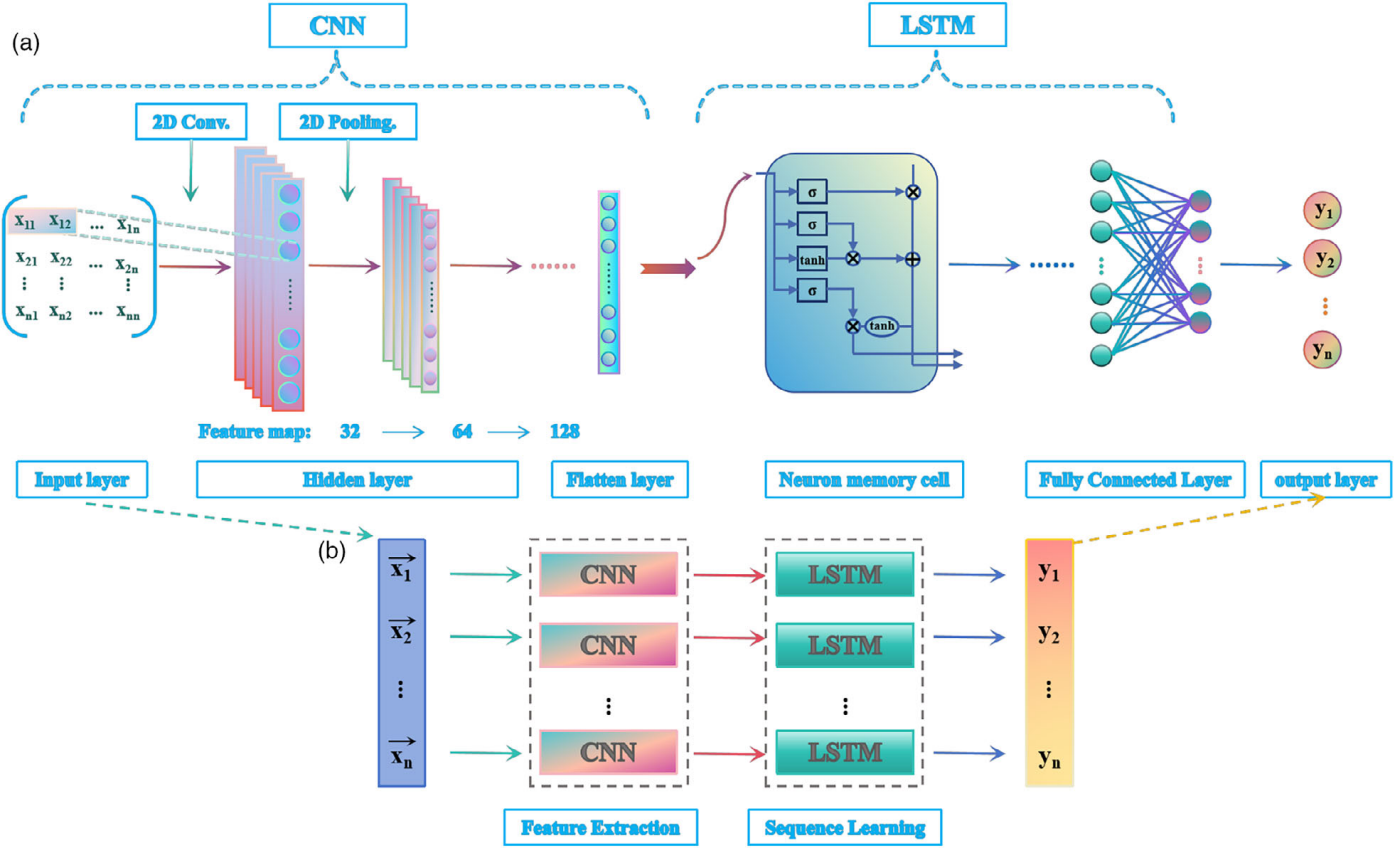
The CNN‐LSTM framework. (a) The overall framework of CNN‐LSTM; (b) The running order of CNN‐LSTM.

As shown in Figure 7, the CNN‐LSTM framework was constructed. In this case, the CNN model contains three modules, the input layer, the hidden layer and the output layer. The acquired hyperspectral data were used as the input to the CNN model. The data were preprocessed to reduce the effect of anomalous data on the model. Convolution, pooling and fully linked layers make up the majority of the hidden layer. Convolutional computation was performed on a 2D data matrix by a convolution kernel of size 1 × 2 with a step size of 2. The maximum pooling layer replaced a point of the feature map with a global feature of its neighbouring outputs for data dimensionality reduction through the pooling function. Through three convolution and pooling operations, 32, 64 and 128 feature maps were obtained respectively. The neuron weights were then connected through the fully connected layer and the data information was passed to the next layer of the network. That is, the weighted summation of the feature vectors was computed by matrix multiplication and the output of the fully connected layer was obtained through the activation function. Finally, a regression layer was added and the output values of the fully connected layer were fed into the regression layer to obtain the final output of the neural network, that is, the result of the nonlinear transformation of the nonlinear mapping of the neural network.

LSTM is a modified recurrent neural network (RNN), usually with a chain structure. A gate structure that stores the neuronal states is included in the LSTM, which makes it distinctive. The hidden layer of the RNN was given a forget gate, input gate and output gate structure by the LSTM, which allowed it to receive the output of the neurons in the preceding layer and, via the gate structure, preferentially preserve important information from past moments. The output of the hidden layer of the CNN model was taken as the input to the LSTM. The input was computed by the input gate. If the output was approximate to zero, the value here would be blocked and would not proceed to the next layer. Then, it passed through the forget gate, when this generated value was approximate to zero, the value remembered in the block would be forgotten. Finally, it passed through the output gate, this layer decided whether the information remembered in the block can be output or not.

Combining the CNN model with the LSTM model has taken into account the advantages of the two models in extracting feature information from high‐dimensional data and processing time series data respectively. To achieve the prediction of SOD activity in tomato leaves, the LSTM model architecture was embedded after the hidden layer of the CNN model. The flattened layer of the CNN model was used as the input of the LSTM model, and the output layer of the LSTM was flattened as the fully connected layer, and the final prediction was obtained after the regression layer. The results of the output layer were presented in terms of the correlation coefficient (R) and root mean square error (RMSE) of the model predictions.

### Transfer learning

Transfer learning allows new problems to be solved quickly using previously learnt knowledge. In order to create prediction functions, it gathers the knowledge acquired from the source tasks and source domain. The capacity of transfer learning to provide multi‐domain learning enables the relationship between many activities or domains utilized in the source and destination. A few academics have started looking at CNN‐based transfer learning in recent years. Model transfer can serve as a concise summary of the study's overall transfer learning concept. The optimal CNN‐LSTM model based on macroscopic spectra was combined with H2D‐COS analysis to find the migration wavelengths with strong correlation for transfer learning. Optimal macroscopic spectral prediction models were transferred to microscopic models for macro‐ and micro‐correlation.

## Author contributions

**Jie Hao:** Writing–original draft; Methodology, Investigation, Data curation. **Yan Yan:** writing–review and editing. **Yao Zhang:** writing–review and editing. **Yiyang Zhang:** Supervision, Formal analysis. **Yune Cao:** writing–review and editing, Funding acquisition. **Longguo Wu:** Project administration, Investigation, Funding acquisition, Conceptualization.

## Conflict of interest

The authors declare no conflict of interest.

## Supporting information


**Figure S1** The microhyperspectral curves pre‐treated by different methods. (a) Raw spectral curves; (b) Smoothing; (c) Baseline; (d) FD.
**Figure S2** The model performance of the microhyperspectral data with different preprocessing.

## Data Availability

The data that support the findings of this study are available on request from the corresponding author. The data are not publicly available due to privacy or ethical restrictions.

## References

[pbi14566-bib-0001] Acosta‐Motos, J. , Ortuño, M. , Bernal‐Vicente, A. , Diaz‐Vivancos, P. , Sanchez‐Blanco, M. and Hernandez, J. (2017) Plant responses to salt stress: Adaptive mechanisms. Agronomy‐Basel 7, 18.

[pbi14566-bib-0002] Al‐Sarayreh, M. , Reis, M.M. , Yan, W.Q. and Klette, R. (2020) Potential of deep learning and snapshot hyperspectral imaging for classification of species in meat. Food Control. 117, 107332.

[pbi14566-bib-0003] Ashraf, M.A. , Akbar, A. , Parveen, A. , Rasheed, R. , Hussain, I. and Iqbal, M. (2018) Phenological application of selenium differentially improves growth, oxidative defense and ion homeostasis in maize under salinity stress. Plant Physiol. Biochem. 123, 268–280. 29275208 10.1016/j.plaphy.2017.12.023

[pbi14566-bib-0004] Bai, Z. , Hu, X. , Tian, J. , Chen, P. , Luo, H. and Huang, D. (2020) Rapid and nondestructive detection of sorghum adulteration using optimization algorithms and hyperspectral imaging. Food Chem. 331, 127290. 32544654 10.1016/j.foodchem.2020.127290

[pbi14566-bib-0005] Bankaji, I. , Sleimi, N. , Vives‐Peris, V. , Gómez‐Cadenas, A. and Pérez‐Clemente, R.M. (2019) Identification and expression of the Cucurbita WRKY transcription factors in response to water deficit and salt stress. Sci. Hortic. 256, 108562.

[pbi14566-bib-0006] Barbon, A.P.A.C. , Barbon, S. , Mantovani, R.G. , Fuzyi, E.M. , Peres, L.M. and Bridi, A.M. (2016) Storage time prediction of pork by Computational Intelligence. Comput. Electro. Agr. 127, 368–375.

[pbi14566-bib-0007] Biswas, S. , Ghoshal, D. and Hazra, R. (2016) A new algorithm of image segmentation using curve fitting based higher order polynomial smoothing. Optik 127, 8916–8925.

[pbi14566-bib-0008] Cheng, W. , Sun, D.W. , Pu, H. and Wei, Q. (2018) Heterospectral two‐dimensional correlation analysis with near‐infrared hyperspectral imaging for monitoring oxidative damage of pork myofibrils during frozen storage. Food Chem. 248, 119–127. 29329834 10.1016/j.foodchem.2017.12.050

[pbi14566-bib-0009] Cui, J. , Yang, M. , Son, D. , Cho, S. and Kim, G. (2020) Hyperspectral Imaging for Tomato Bruising Damage Assessment of Simulated Harvesting Process Impact Using Wavelength Interval Selection and Multivariate Analysis. Appl. Eng. Agric. 36, 533–547.

[pbi14566-bib-0010] Fang, H. , Zou, Q. , He, Y. and Li, X. (2012) Detection of Activity of POD in Tomato Leaves Based on Hyperspectral Imaging Technology. Spectrosc. Spect. Anal. 32, 2228–2233. 23156787

[pbi14566-bib-0011] Hao, J. , Dong, F. , Li, Y. , Wang, S. , Cui, J. , Zhang, Z. and Wu, K. (2022) Investigation of the data fusion of spectral and textural data from hyperspectral imaging for the near geographical origin discrimination of wolfberries using 2D‐CNN algorithms. Infrared Phys. Techn. 125, 104286.

[pbi14566-bib-0012] Kang, R. , Park, B. , Eady, M. , Qin, O. and Chen, K. (2020) Single‐cell classification of foodborne pathogens using hyperspectral microscope imaging coupled with deep learning frameworks. Sensor Actuat B‐Chem. 309, 127789.

[pbi14566-bib-0013] Kim, H.J. , Kim, S.B. , Kim, J.K. and Jung, Y.M. (2006) Two‐dimensional heterospectral correlation analysis of wide‐angle X‐ray scattering and infrared spectroscopy for specific chemical interactions in weakly interacting block copolymers. J. Phys. Chem. B 110, 23123–23129. 17107153 10.1021/jp0638282

[pbi14566-bib-0014] Kong, W. , Liu, F. , Zhang, C. , Bao, Y. , Yu, J. and He, Y. (2014) Fast detection of peroxidase (POD) activity in tomato leaves which infected with Botrytis cinerea using hyperspectral imaging. Spectrochim. Acta A 118, 498–502. 10.1016/j.saa.2013.09.009 24080581

[pbi14566-bib-0015] Le, T. , Liu, C. , Yao, B. , Natraj, V. and Yung, Y.L. (2020) Application of machine learning to hyperspectral radiative transfer simulations. J. Quant. Spectrosc. Ra. 246, 106928.

[pbi14566-bib-0016] Lin, Y. , Qiu, H. , Lu, Y. , Chen, G. , Lin, Y. , Guo, W. , Jin, J. *et al*. (2019) Determining Temperature‐Dependent Optical Characteristics of Remote Phosphor Plates by Transmission‐Type Measurement System. IEEE T Electron. Dev. 66, 1322–1328.

[pbi14566-bib-0017] Ma, T. , Tsuchikawa, S. and Inagaki, T. (2020) Rapid and non‐destructive seed viability prediction using near‐infrared hyperspectral imaging coupled with a deep learning approach. Comput. Electro. Agr. 177, 105683.

[pbi14566-bib-0018] Maqbool, T. , Ly, Q.V. , Asif, M.B. , Ng, H.Y. and Zhang, Z. (2020) Fate and role of fluorescence moieties in extracellular polymeric substances during biological wastewater treatment: A review. Sci. Total Environ. 718, 137291. 32087584 10.1016/j.scitotenv.2020.137291

[pbi14566-bib-0019] Mehlmer, N. , Wurzinger, B. , Stael, S. , Hofmann‐Rodrigues, D. , Csaszar, E. , Pfister, B. , Bayer, R. *et al*. (2010) The Ca^2+^‐dependent protein kinase CPK3 is required for MAPK‐independent salt‐stress acclimation in Arabidopsis. Plant J. 63, 484–498. 20497378 10.1111/j.1365-313X.2010.04257.x PMC2988408

[pbi14566-bib-0020] Peskin, A.V. and Winterbourn, C.C. (2017) Assay of superoxide dismutase activity in a plate assay using WST‐1. Free Radic. Biol. Med. 103, 188–191. 28017897 10.1016/j.freeradbiomed.2016.12.033

[pbi14566-bib-0021] Qi, L. , Li, J. , Wang, Y. , Lei, M. and Gao, X. (2020) Deep spectral convolution network for hyperspectral image unmixing with spectral library. Signal Process. 176, 107672.

[pbi14566-bib-0022] Qin, O. , Yang, Y. , Park, B. , Kang, R. , Wu, J. , Chen, Q. , Guo, Z. *et al*. (2020) A novel hyperspectral microscope imaging technology for rapid evaluation of particle size distribution in matcha. J. Food Eng. 272, 109782.

[pbi14566-bib-0023] Qin, J. , Vasefi, F. , Hellberg, R.S. , Akhbardeh, A. , Isaacs, R.B. , Yilmaz, A.G. , Hwang, C. *et al*. (2020) Detection of fish fillet substitution and mislabeling using multimode hyperspectral imaging techniques. Food Control. 114, 107234.

[pbi14566-bib-0024] Ramos, S. , Segovia, L. , Melado‐Herreros, A. , Cidad, M. , Zufia, J. , Vranken, L. and Matthys, C. (2022) Enviroscore: normalization, weighting, and categorization algorithm to evaluate the relative environmental impact of food and drink products. NPJ Sci. Food. 6, 54. 36433991 10.1038/s41538-022-00165-z PMC9700787

[pbi14566-bib-0025] Rodrigues, F.J. , Blasch, G. , Defourny, P. , Ortiz‐Monasterio, J.I. , Schulthess, U. , Zarco‐Tejada, P.J. , Taylor, J.A. *et al*. (2018) Multi‐Temporal and Spectral Analysis of High‐Resolution Hyperspectral Airborne Imagery for Precision Agriculture: Assessment of Wheat Grain Yield and Grain Protein Content. Remote Sens‐Basel 10, 930. 32704487 10.3390/rs10060930 PMC7340494

[pbi14566-bib-0026] Singh, M. , Kumar, J. , Singh, S. , Singh, V.P. and Prasad, S.M. (2015) Roles of osmoprotectants in improving salinity and drought tolerance in plants: a review. Rev. Environ. Sci. Bio. 14, 407–426.

[pbi14566-bib-0027] Sun, Y. , Pessane, I. , Pan, L. and Wang, X. (2021) Hyperspectral characteristics of bruised tomatoes as affected by drop height and fruit size. LWT. 141, 110863.

[pbi14566-bib-0028] Takashi, N. , Takashi, G. , Masahiro, W. and Yukihiro, O. (2013) Selection of the NIR region for a regression model of the ethanol concentration in fermentation process by an online NIR and Mid‐IR dual‐region spectrometer and 2D heterospectral correlation spectroscopy. Anal. Sci. 28, 1165–1170. 10.2116/analsci.28.1165 23232236

[pbi14566-bib-0029] Tang, J. , Zhuang, L. , Yu, Z. , Liu, X. , Wang, Y. , Wen, P. and Zhou, S. (2019) Insight into complexation of Cu(II) to hyperthermophilic compost‐derived humic acids by EEM‐PARAFAC combined with heterospectral two dimensional correlation analyses. Sci. Total Environ. 656, 29–38. 30502732 10.1016/j.scitotenv.2018.11.357

[pbi14566-bib-0030] Taniguchi, N. , Maeda, K. and Kitano, M. (2022) On ‘Generation of superoxide radical during autoxidation of hydroxylamine and an assay for superoxide dismutase’ by Yasuhisa Kono. Arch. Biochem. Biophys. 726, 109115. 34986418 10.1016/j.abb.2021.109115

[pbi14566-bib-0031] Wan, L. , Zhou, W. , He, Y. , Wanger, T. and Cen, H. (2022) Combining transfer learning and hyperspectral reflectance analysis to assess leaf nitrogen concentration across different plant species datasets. Remote Sens. Environ. 269, 112826.

[pbi14566-bib-0032] Wang, S. , Guan, K. , Wang, Z. , Ainsworth, E.A. , Zheng, T. , Townsend, P.A. , Liu, N. *et al*. (2021) Airborne hyperspectral imaging of nitrogen deficiency on crop traits and yield of maize by machine learning and radiative transfer modeling. Int. J. Appl. Earth Obs. 105, 102617.

[pbi14566-bib-0033] Wu, C.Q. and Chen, G.H. (2019) Detection of synergistic effect of superoxide dismutase and jujubosides on scavenging superoxide anion radical by capillary electrophoresis. Biomed. Chromatogr. 33, e4630. 31243785 10.1002/bmc.4630

[pbi14566-bib-0035] Xiang, Y. , Chen, Q. , Su, Z. , Zhang, L. , Chen, Z. , Zhou, G. , Yao, Z. *et al*. (2022) Deep Learning and Hyperspectral Images Based Tomato Soluble Solids Content and Firmness Estimation. Front. Plant Sci. 13, 860656. 35586212 10.3389/fpls.2022.860656 PMC9108868

[pbi14566-bib-0036] Xiao, Q. , Tang, W. , Zhang, C. , Zhou, L. , Feng, L. , Shen, J. , Yan, T. *et al*. (2022) Spectral preprocessing combined with deep transfer learning to evaluate chlorophyll content in cotton leaves. Plant Phenomics 2022, 9813841. 36158530 10.34133/2022/9813841 PMC9489230

[pbi14566-bib-0037] Yang, R. , Liu, R. , Dong, G. , Xu, K. , Yang, Y. and Zhang, W. (2016) Two‐dimensional hetero‐spectral mid‐infrared and near‐infrared correlation spectroscopy for discrimination adulterated milk. Spectrochim. Acta A 157, 50–54. 10.1016/j.saa.2015.12.017 26714285

[pbi14566-bib-0038] Zarco‐Tejada, P.J. , González‐Dugo, V. and Berni, J.A.J. (2012) Fluorescence, temperature and narrow‐band indices acquired from a UAV platform for water stress detection using a micro‐hyperspectral imager and a thermal camera. Remote Sens. Environ. 117, 322–337.

[pbi14566-bib-0039] Zhang, J. and Li, S. (2022) Air quality index forecast in Beijing based on CNN‐LSTM multi‐model. Chemosphere 308, 136380. 36058367 10.1016/j.chemosphere.2022.136180

[pbi14566-bib-0040] Zhang, X. , Liu, F. , He, Y. and Gong, X. (2013) Detecting macronutrients content and distribution in oilseed rape leaves based on hyperspectral imaging. Biosyst Eng. 115, 56–65.

[pbi14566-bib-0041] Zhang, C. , Wu, W. , Zhou, L. , Cheng, H. , Ye, X. and He, Y. (2020) Developing deep learning based regression approaches for determination of chemical compositions in dry black goji berries (Lycium ruthenicum Murr.) using near‐infrared hyperspectral imaging. Food Chem. 319, 126536. 32146292 10.1016/j.foodchem.2020.126536

[pbi14566-bib-0042] Zhang, Y. , Hui, J. , Qin, Q. , Sun, Y. , Zhang, T. , Sun, H. and Li, M. (2021) Transfer‐learning‐based approach for leaf chlorophyll content estimation of winter wheat from hyperspectral data. Remote Sens. Environ. 267, 112724.

[pbi14566-bib-0043] Zhang, L. , Zhang, Q. , Wu, J. , Liu, Y. , Yu, L. and Chen, Y. (2022) Moisture detection of single corn seed based on hyperspectral imaging and deep learning. Infrared Phys Techn. 125, 104279.

[pbi14566-bib-0044] Zhang, C. , Li, C. , He, M. , Feng, Z. , Qi, H. and Zhou, H. (2023) Leaf water content determination of oilseed rape using near‐infrared hyperspectral imaging with deep learning regression methods. Infrared Phys Techn. 134, 104921.

[pbi14566-bib-0045] Zhou, X. , Sun, J. , Tian, Y. , Yao, K. and Xu, M. (2022) Detection of heavy metal lead in lettuce leaves based on fluorescence hyperspectral technology combined with deep learning algorithm. Spectrochim. Acta A 266, 120460. 10.1016/j.saa.2021.120460 34637985

[pbi14566-bib-0046] Zhou, X. , Zhao, C. , Sun, J. , Yao, K. and Xu, M. (2023) Detection of lead content in oilseed rape leaves and roots based on deep transfer learning and hyperspectral imaging technology. Spectrochim. Acta A 290, 122288. 10.1016/j.saa.2022.122288 36608517

[pbi14566-bib-0047] Zhou, X. , Zhao, C. , Sun, J. , Cheng, J. and Xu, M. (2024) Determination of lead content in oilseed rape leaves in silicon‐free and silicon environments based on deep transfer learning and fluorescence hyperspectral imaging. Spectrochim. Acta A 311, 123991. 10.1016/j.saa.2024.123991 38330763

[pbi14566-bib-0048] Zhu, W. , Li, J. , Li, L. , Wang, A. , Wei, X. and Mao, H. (2020) Nondestructive diagnostics of soluble sugar, total nitrogen and their ratio of tomato leaves in greenhouse by polarized spectra ‐ hyperspectral data fusion. Int. J. Agric. Biol. Eng. 13, 189–197.

