# Supplementary material for: A cross‐scale transfer learning framework: prediction of SOD activity from leaf microstructure to macroscopic hyperspectral imaging
Source: Plant Biotechnol J. 2025 Jan 9;23(4):1091–100. doi: 10.1111/pbi.14566 (PMC11933873; doi:10.1111/pbi.14566)
Supplement: Supplementary file 1 — Figure S1 The microhyperspectral curves pre‐treated by different methods. (a) Raw spectral curves; (b) Smoothing; (c) Baseline; (d) FD. Figure S2 The model performance of the microhyperspectral data with different preprocessing. [file PBI-23-1091-s001.docx]

**A cross-scale transfer learning framework: prediction of SOD activity from leaf microstructure to macroscopic hyperspectral imaging**

Jie Hao ^a,c,1^, Yan Yan^f,1^, Yao Zhang ^b,d,1^, Yiyang Zhang ^a^, Yune Cao ^a,**^, Longguo Wu ^a,e,*^

*a School of Wine & Horticulture, Ningxia University, Yinchuan, Ningxia, 750021, China*

*b College of Animal Science and Technology, Ningxia University, Yinchuan, Ningxia, 750021, China*

*c College of Mechanical and Electronic Engineering, Northwest A&F University, Yangling, Shaanxi 712100, China*

*d Key Laboratory of Quality and Safety of Wolfberry and Wine for State Administration for Market Regulation, Institute of Food Testing in Ningxia, Yinchuan, Ningxia, 750021, China*

*e Ningxia Modern Protected Horticulture Engineering Technology Research Center, Yinchuan, Ningxia, 750021, China*

*^f^ State Key Laboratory of Vegetable Biobreeding, Institute of Vegetables and Flowers, Chinese Academy of Agricultural Sciences, Beijing 100081, China*

Jie Hao, Yan Yan and Yao Zhang have equal contribution.

*Corresponding author: [wlg@nxu.edu.cn](mailto:wlg@nxu.edu.cn).

**Corresponding author: caohua3221@163.com.

**Fig. S1.** The microhyperspectral curves pre-treated by different methods. (a) Raw spectral curves; (b) Smoothing; (c) Baseline; (d) FD.

**Fig. S2.** The model performance of the microhyperspectral data with different preprocessing.
